# Quantitative phosphoproteomic analysis of chicken DF-1 cells infected with *Eimeria tenella*, using tandem mass tag (TMT) and parallel reaction monitoring (PRM) mass spectrometry[Fn FN1]

**DOI:** 10.1051/parasite/2024027

**Published:** 2024-05-17

**Authors:** Liu-Shu Jia, Zhan Liu, Shun-Hai Zhu, Qi-Ping Zhao, Hong-Yu Han, Huan-Zhi Zhao, Yu Yu, Hui Dong

**Affiliations:** Shanghai Veterinary Research Institute, Chinese Academy of Agricultural Sciences, Key Laboratory of Animal Parasitology of Ministry of Agriculture Minhang Shanghai 200241 PR China

**Keywords:** Coccidiosis, *Eimeira tenella*, DF-1 cells, TMT, Phosphoproteomics

## Abstract

*Eimeria tenella* is an obligate intracellular parasite which causes great harm to the poultry breeding industry. Protein phosphorylation plays a vital role in host cell–*E. tenella* interactions. However, no comprehensive phosphoproteomic analyses of host cells at various phases of *E. tenella* infection have been published. In this study, quantitative phosphoproteomic analysis of chicken embryo DF-1 fibroblasts that were uninfected (UI) or infected with *E. tenella* for 6 h (PI6, the early invasion phase) or 36 h (PI36, the trophozoite development phase) was conducted. A total of 10,122 phosphopeptides matched to 3,398 host cell phosphoproteins were identified and 13,437 phosphorylation sites were identified. Of these, 491, 1,253, and 275 differentially expressed phosphorylated proteins were identiﬁed in the PI6/UI, PI36/UI, and PI36/PI6 comparisons, respectively. KEGG pathway enrichment analysis showed that *E. tenella* modulated host cell processes through phosphorylation, including focal adhesion, regulation of the actin cytoskeleton, and FoxO signaling to support its early invasion phase, and modulating adherens junctions and the ErbB signaling pathway to favor its trophozoite development. These results enrich the data on the interaction between *E. tenella* and host cells and facilitate a better understanding of the molecular mechanisms underlying host–parasite relationships.

## Abstract (fr)

*Eimeria tenella* est un parasite intracellulaire obligatoire qui cause de graves dommages à l’industrie de l’élevage de volailles. La phosphorylation des protéines joue un rôle essentiel dans les interactions entre la cellule hôte et *E. tenella*. Cependant, aucune analyse phosphoprotéomique complète des cellules hôtes à différentes phases de l’infection par *E. tenella* n’a été publiée. Dans cette étude, une analyse phosphoprotéomique quantitative de fibroblastes DF-1 d’embryon de poulet non infectés (NI) ou infectés par *E. tenella* pendant 6 h (PI6, la phase d’invasion précoce) ou 36 h (PI36, la phase de développement des trophozoïtes) a été réalisée. Un total de 10 122 phosphopeptides correspondant à 3 398 phosphoprotéines de cellules hôtes ont été identifiés et 13 437 sites de phosphorylation ont été identifiés. Parmi celles-ci, 491, 1 253 et 275 protéines différentiellement phosphorylées exprimées ont été identifiées respectivement dans les comparaisons PI6/NI, PI36/NI et PI36/PI6. L’analyse d’enrichissement de la voie KEGG a montré qu’*E. tenella* modulait les processus de la cellule hôte par phosphorylation, y compris l’adhésion focale, la régulation du cytosquelette d’actine et la signalisation FoxO, pour aider sa phase d’invasion précoce, et la modulation des jonctions adhérentes et de la voie de signalisation ErbB pour favoriser le développement de son trophozoïte. Ces résultats enrichissent les données sur l’interaction entre *E. tenella* et les cellules hôtes et facilitent une meilleure compréhension des mécanismes moléculaires sous-jacents aux relations hôtes–parasites.

## Introduction

Chicken coccidiosis is caused by the genus *Eimeria* and is one of the most common and devastating parasitic diseases in the poultry industry [3]. It can damage the intestinal epithelial cells of chickens, hinder the absorption of nutrients, and reduce the production of meat and eggs, causing economic losses of approximately £10.4 billion annually worldwide [4]. Currently, the prevention and control methods of coccidiosis mainly include anticoccidial drugs and live vaccines. However, these methods raise several issues ranging from drug resistance to food security and production costs [6, 39]. Therefore, it is necessary to find novel measures to control coccidiosis.

*Eimeria* relies on host cells to complete its life cycle. In the process of *Eimeria* infection, some physiological and biochemical processes of host cells are regulated, supporting the invasion and development of the parasite. For instance, invasion of host cells by *Eimeria tenella* causes host F-actin aggregation on the sporozoite surface. This aggregation is closely related to the invasion efficiency of *E. tenella*, and inhibition of F-actin aggregation by cytochalasin D reduces the invasion rate of *E. tenella* [47]. The expression of host fatty acid binding protein 4 increases significantly at 72 h after invasion of *E. tenella* sporozoites, and overexpression of this protein significantly inhibits the invasion of *E. tenella* sporozoites [45]. Incubation of host cells with polyclonal antibodies targeting receptors for activated C kinase 1 (RACK1) can promote the invasion of *E. tenella* sporozoites [51]. The vimentin transcript and protein levels increase continually at 6–72 h after *E. tenella* infection, and treating DF-1 cells with polyclonal anti-vimentin or knocking down vimentin by small interfering RNA significantly improves the invasion efficiency of sporozoites, indicating that vimentin plays an inhibitory role during the invasion of sporozoites [31]. These results demonstrate that host proteins play an indispensable role in *Eimeria* invasion and intracellular development. However, the extent and relevance of *Eimeria*-induced host responses remain poorly studied.

Many studies have confirmed that protein phosphorylation is involved in the interaction between pathogenic microorganisms and host cells [11, 18]. Phosphorylation is one of the most common post-translational modifications in organisms and plays an important role in cell growth, differentiation, apoptosis, and cell signaling in healthy conditions [41]. It occurs mainly on serine, threonine, or tyrosine residues and can affect the function of one third of the proteins [9]. Studies have found that the PI3K–PKB/Akt pathway may be one of the major routes through which *Toxoplasma gondii* prevents host cell apoptosis and that *T. gondii* phosphorylates the pro-apoptotic protein bad to prevent apoptosis [36]. So far, no studies on host cell phosphoproteomics after *E. tenella* infection have been published.

In recent years, tandem mass tag (TMT)-labeled quantitative proteomics technology coupled with high-resolution mass spectrometry (MS) has been proven to be an efficient technique for proteome studies simultaneously identifying and comparing the relative contents of proteins in up to 10 different samples. It has been widely used in the study of phosphoproteomics [29]. In the present study, the phosphoproteomes of DF-1 cells before and after *E. tenella* infection were comparatively analyzed by TMT-labeled quantitative proteomics. The data in this study are helpful to identify key host proteins associated with *E. tenella* invasion and provide an important basis to further analyze *E. tenella*–host cell interactions.

## Materials and methods

### Parasite, cell culture and ethics statement

The *E. tenella* Shanghai strain used in the current study has been maintained in our laboratory since 1993. *E. tenella* was propagated by passage through 14-day-old coccidia-free chickens [43]. Sporozoites were collected and purified from cleaned sporulated oocysts with standard procedures [17, 50]. DF-1 cells were cultured in Dulbecco’s modified Eagle’s medium (DMEM) (Life Technology, Carlsbad, CA, USA) supplemented with 10% fetal bovine serum at 37 °C in a 5% CO_2_ incubator.

The animal protocols were approved by the Institutional Animal Care and Use Committee of Shanghai Veterinary Research Institute, Chinese Academy of Agricultural Sciences (Permit Number: SHVRI-SZ-20230323-4).

### Parasite infection and sample collection

DF-1 cells were seeded into T25 culture flasks (1 × 10^6^ cells per flask) and allowed to reach 80–90% confluence before infection. Freshly excysted sporozoites were incubated with DMEM supplemented with 2% fetal bovine serum and 5% penicillin/streptomycin at 37 °C for 2 h. DF-1 cells were infected with pre-treated sporozoites at a multiplicity of infection of 3 for 6 h (6 h post-infection, PI6) or 36 h (36 h post-infection, PI36). Uninfected (UI) cells were used as control. Each experiment was performed with three independent biological replicates.

At 6 h after infection, the cell culture supernatant containing the sporozoites was removed and the non-invasive sporozoites were removed by washing gently with PBS three times. Cells in the UI and PI6 groups were harvested with a cell scraper; cells in the PI36 group were cultured with complete DMEM for another 30 h and then harvested with a cell scraper. All cells were pelleted by centrifugation and stored at −80 °C for subsequent analysis.

### Protein extraction and peptide enzymatic hydrolysis

The cell samples were lysed with SDT buffer (4% SDS, 100 mM Tris/HCl pH 7.6, and 0.1 M DTT), incubated for 3 min in boiling water, sonicated on ice twice, and clariﬁed by centrifugation at 16,000 ×*g* at 25 °C for 10 min. Protein content was quantified with a BCA Protein Assay Kit (Bio-Rad, Hercules, CA, USA).

A moderate amount of protein was extracted from each sample, and trypsin enzymatic hydrolysis was performed using the filter-aided sample preparation method [49], followed by desalting enzymolysis of peptides using a C18 Cartridge. Lyophilized peptides were dissolved with 40 μL dissolution buffer and the peptides were quantified by measuring the optical density at 280 nm.

### TMT labeling and SCX chromatographic classification

First, 100 μg peptide mixture was taken from each sample and labeled using a TMT labeling kit, according to the manufacturer’s instructions (Thermo Fisher, Waltham, MA, USA). The labeled peptides were fractionated by strong cation exchange (SCX) liquid chromatography (LC) using an AKTA Purifier 100 system. Gradient elution was performed using buffer A (10 mM KH_2_PO_4_, 25% acetonitrile, pH 3.0) and buffer B (10 mM KH_2_PO_4_, 500 mM KCl, 25% acetonitrile, pH 3.0). The chromatographic column was balanced using buffer A and the sample was applied to the column for separation. The flow rate was 1 mL/min and the gradient elution program was as follows: 0–25 min, 0–10% buffer B; 25–32 min, 10–20% buffer B; 32–42 min, 20–45% buffer B; 42–47 min, 45–100% buffer B; 47–60 min, 100% buffer B; 60 min, 0% buffer B. The absorbance at 214 nm was monitored during elution, fractions were collected every 1 min, and the collected fractions were desalted with a C18 Cartridge [23].

### TiO_2_ enrichment of phosphopeptides

The labeled peptide mixtures were vacuum-freeze dried and dissolved with 1× DHB buffer [obtained by diluting 5× DHB buffer (3% dihydrobenzoic acid, 80% acetonitrile, 0.1% trifluoroacetic acid) with water at a 1:4 ratio]. Next, TiO_2_ beads were added to the solution, samples were oscillated for 40 min and centrifuged, and the supernatant was removed. The precipitates were washed three times with wash buffer I (30% acetonitrile and 3% trifluoroacetic acid) and three times with washing buffer II (80% acetonitrile and 0.3% trifluoroacetic acid). The enriched phosphorylated peptides were eluted with elution buffer (40% acetonitrile and 15% NH_4_OH) and then vacuum concentrated. The phosphorylated peptides were redissolved in 10 μL of 0.1% formic acid, and 5 μL was taken for MS analysis.

### LC-MS/MS analysis

Each sample was analyzed by a nano-scale high-performance liquid phase system (EASY-nLC) using buffer A (0.1% aqueous formic acid solution) and buffer B (0.1% aqueous acetonitrile solution). The chromatographic column was balanced using 95% of buffer A, and then sample was added to the sample column (Thermo Scientific EASY column, 100 μm × 2 cm, 5 μm, C18) and separated by analytical columns (Thermo Scientific EASY column, 75 μm × 10 cm, 3 μm, C18). The flow rate was 250 nL/min, and the gradient program was as follows: 0–50 min, 0–35% buffer B; 50–58 min, 35–100% buffer B; 58–60 min, 100% buffer B.

After chromatographic separation, the samples were analyzed by LC-MS/MS using a Q-Exactive HF-X mass spectrometer. The analysis time was 120 min, detection was performed in positive ion mode, and the scanning range of parent ions was 300–1,800 *m*/*z*. The resolution of the first-order mass spectrum was 70,000 at *m*/*z* 200, the automatic control target was set at 3e6, and the maximum injection time was set to 10 ms. The dynamic exclusion time was 40.0 s. Ten MS2 scan debris maps were collected after each full scan. The activation type of MS2 was high collision dissociation, and the isolation window was 2 *m*/*z*. The resolution of secondary MS was 17,500 at *m*/*z* 200. The normalized collision energy was 30 eV and the underfill ratio was set to 0.1%.

### Phosphoprotein identification and quantitative analysis

Raw data were processed by Proteome Discoverer (Version 1.4, Thermo Electron, San Jose, CA, USA) and matched against the UniProt *Gallus gallus* database (20230902, 35,124 sequences) by scoring through the Mascot server (Version 2.3, Matrix Science, London, UK). Data were searched using the following parameters: peptide mass tolerance = 20 ppm; enzyme = trypsin; fragment mass tolerance = 0.1 Da; ﬁxed modiﬁcation = TMT 10plex (N-term), carbamidomethyl (C), TMT 10plex (K); variable modiﬁcation = oxidation (M), TMT 10plex (Y), deamidated (NQ), phosphorylation (S/T/Y); max missed cleavage = 2. The incorporated Target Decoy PSM Validator in Proteome Discoverer was used to validate the search results with only the hits with a false discovery rate of ≤0.01. The protein ratios were calculated as the median of only unique peptides of the protein. All peptide ratios were normalized by the median protein ratio. The median protein ratio should be 1 after normalization. A phosphoprotein showing a median fold change of >1.5 or <0.67 in phosphorylation level within the group at *p* ≤ 0.05 was considered to be a signiﬁcantly differentially regulated phosphoprotein.

### Bioinformatics analysis

All significantly differentially regulated phosphoproteins in the three comparisons (PI6/UI, PI36/UI, and PI36/PI6) were subjected to bioinformatics analysis. Blast2GO (https://www.blast2go.com/) was used to for Gene Ontology (GO) annotation and enrichment analysis in the cell component, biological process, and molecular function categories. Kyoto Encyclopedia of Genes and Genomes (KEGG) pathway enrichment analysis was performed using the online KEGG Automatic Annotation Server, and the annotation results were mapped to the KEGG pathway database using KEGG mapper. A two-tailed Fisher exact test was employed to assess the enrichment of proteins with differentially regulated phosphosites (GO, KEGG pathway, and protein domain; *p* < 0.05 was considered significant).

### PRM validation of proteins with differentially regulated phosphosites

Validation of proteomics data by parallel reaction monitoring (PRM) is the latest development in targeted MS, serving as the “golden standard” for protein MS analysis. The differentially expressed phosphorylated proteins (DEPPs) from the results of the TMT analysis were further validated by using PRM analysis. Briefly, the protein samples were prepared by the same method as used for LC-MS/MS analysis. PRM analyses were performed on a Q-Exactive mass spectrometer (Thermo Fisher Scientific). The reverse-phase liquid chromatography (RPLC) fractions of each sample were analyzed using an acquisition method. For each sample, 2 μg of peptides were taken and mixed with 20 fMol of stable isotope internal standard peptide. A total of nine samples were obtained. PRM data analysis was performed using Skyline software (MacCoss Lab, University of Washington, Seattle, WA, USA).

## Results

### Identification and quantification of phosphopeptides

**Figure 1:**
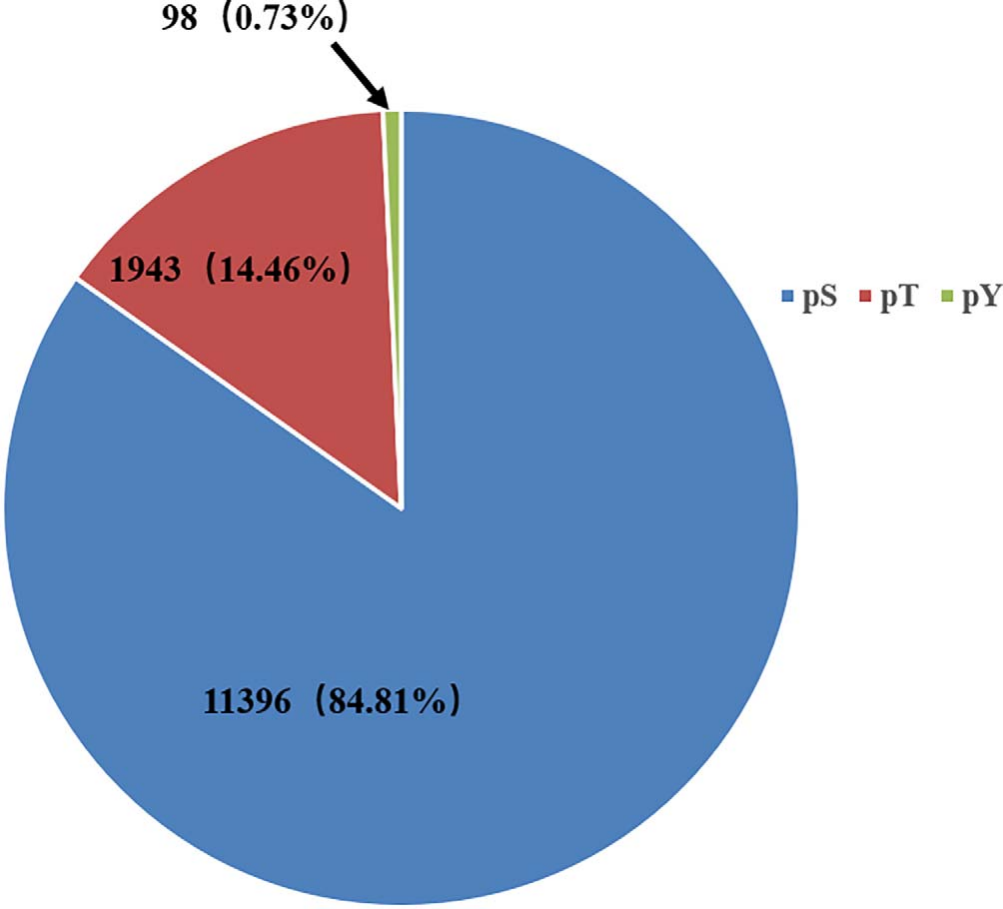
Proportion of serine, threonine, and tyrosine in phosphorylation sites.

The phosphorylated peptides of each group were enriched with TiO_2_ and identified by LC-MS/MS. Through the statistical analysis of raw data, a total of 39,960 peptide spectrum matches (PSMs) were identified and matched to 3,516 host cell proteins (Supplementary Table S1). Among them, 10,122 phosphopeptides (Supplementary Table S2) corresponded to 3,398 host cell phosphoproteins, and 13,437 phosphorylation sites were detected. Among the phosphorylation sites, phospho-serine (pS) was the most abundant, accounting for 84.81% of all the phosphorylated amino acids, followed by phospho-threonine (pT, 14.46%) and phospho-tyrosine (pY, 0.73%) (Figure 1).

### Identification and quantification of the significantly differentially regulated phosphoproteins

To speculate on host proteins that play a role during parasite infection, we analyzed the DEPPs at different time points of infection. The signiﬁcantly differentially expressed phosphorylated peptides were identified based on a fold change value of >1.5 or <0.67 and a *p*-value of <0.05. A comparative phosphorylation level analysis was performed among the UI, PI6, and PI36 groups.

**Figure 2:**
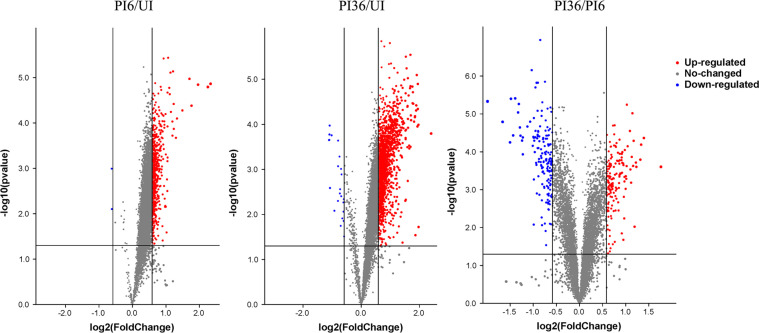
Volcano plots from different group comparisons. The abscissa indicates difference multiple (logarithmic transformation based on 2), the ordinate indicates the significant of difference (logarithmic transformation based on 10). The red point is significantly upregulated phosphorylated peptide segment, the blue point is significantly downregulated phosphorylated peptide segment and the gray point is a phosphorylated peptide segment with no significant difference.

**Figure 3:**
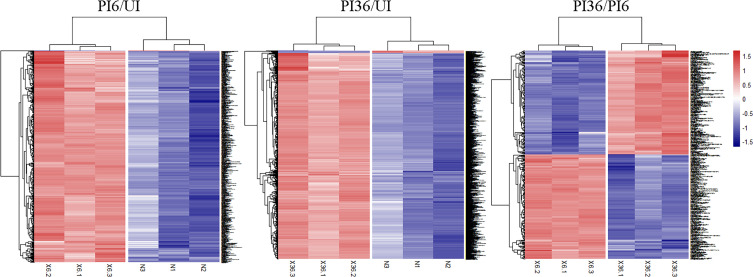
Clustering heatmap of different expression phosphorylated peptides. Each row represents a phosphorylated peptide segment, each column represents a group of samples. The logarithmic value (logarithmic transformation based on 2) of the signiﬁcantly differentially expressed phosphorylated peptides in different samples is displayed in the clustering heatmap in different colors. Red represents significant up-regulation of phosphorylated peptides; blue represents significant down-regulation of phosphorylated peptides.

The comparative analysis led to the identiﬁcation of 489 upregulated and 2 downregulated phosphoproteins in the PI6/UI comparison, 1,228 upregulated and 25 downregulated phosphoproteins in the PI36/UI comparison, and 141 upregulated and 134 downregulated phosphoproteins in the PI36/PI6 comparison (Table 1, Figure 2, Supplementary Table S3). The differentially expressed peptides in the clustering heatmap (Figure 3) show that the protein level trends are consistent between biological replicates in the infected and uninfected groups.

**Table 1:** The number of signiﬁcantly regulated phosphopeptides/proteins among the three comparison groups.

Comparison group	Up-regulated phosphopeptides/proteins	Down-regulated phosphopeptides/proteins	All significantly regulated phosphopeptides/proteins
PI6/UI	731/489	2/2	733/491
PI36/UI	2,189/1,228	27/25	2,216/1,253
PI36/PI6	185/141	186/134	371/275

### GO analysis of differentially expressed phosphoproteins

Fisher’s exact test was used for GO functional enrichment analysis of the DEPPs corresponding to differentially expressed phosphorylated peptides in the three comparisons (PI6/UI, PI36/UI, and PI36/PI6) to identify the top five enriched terms for each comparison.

**Figure 4:**
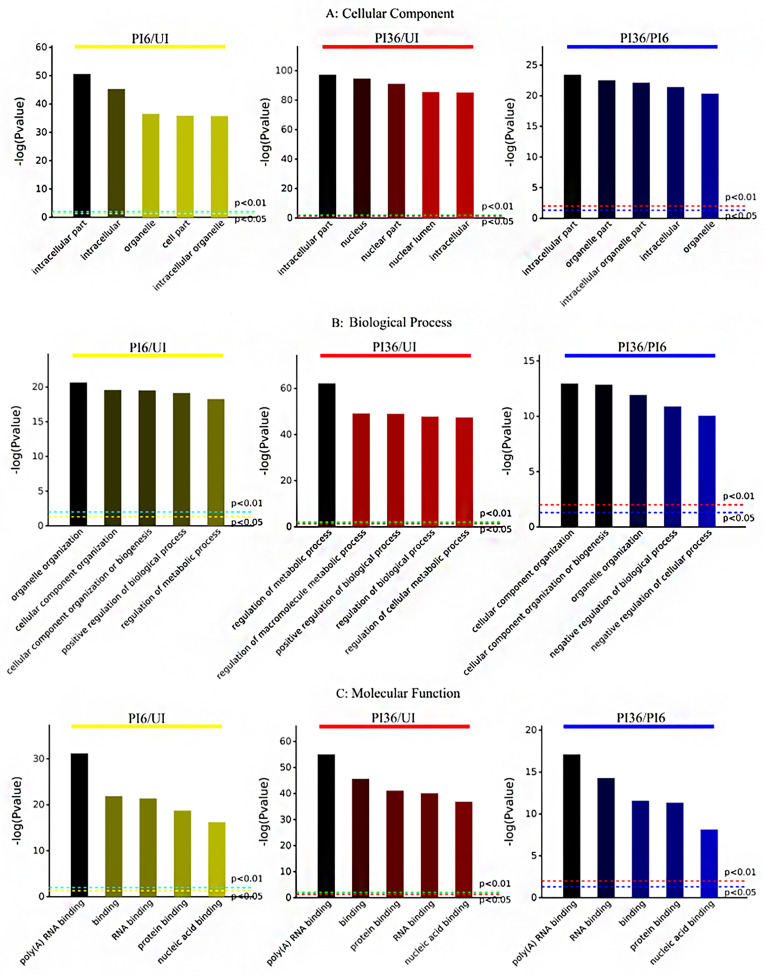
Gene ontology annotations of the differentially expressed phosphorylated proteins. The abscissa indicates the enriched GO functional classification, including biological process (A), cellular component (B), and molecular function (C). The ordinate indicates the size of the significance of corresponding to each entry, the more left, the more significant.

Interestingly, in the cellular component category, the main DEPPs of the three comparisons (PI6/UI, PI36/UI, and PI36/PI6) were all concentrated in the intracellular part. In addition, in the cellular component category, the GO terms were similar between the PI6/UI and PI36/PI6 comparisons, including intracellular, organelle, and intracellular organelle part. In the PI36/UI comparison, the GO terms were mostly related to the nucleus, including nuclear part and nuclear lumen (Figure 4A).

In the biological process category, the DEPPs of PI6/UI and PI36/PI6 were found to be mainly involved in cellular component organization, cellular component organization or biogenesis, and organelle organization. However, it is worth mentioning that we found that metabolic-related biological processes were enriched in the PI36/UI comparison, including regulation of metabolic process, regulation of macromolecule metabolic process, and regulation of cellular metabolic process. Meanwhile, some DEPPs in the PI6/UI comparison were also related to regulation of metabolic process (Figure 4B). These results suggest that sporozoite invasion and intracellular development may depend on the energy provided by host cells.

Additionally, the GO terms in the molecular function category of all three comparisons were associated with binding, including poly(A) RNA binding, RNA binding, binding, and protein binding (Figure 4C). This suggests that both transcription and translation levels were quite active in cells at the parasite infection stage.

### KEGG pathway analysis of differentially expressed phosphoproteins

**Figure 5:**
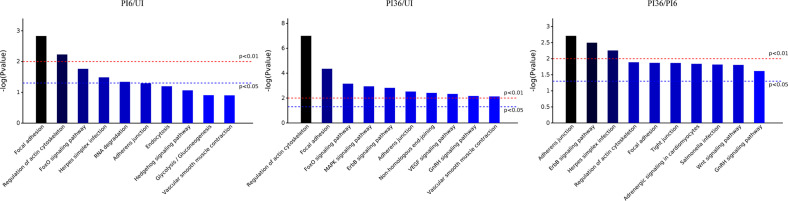
KEGG pathways of the differentially expressed phosphorylated proteins. The abscissa indicates the first 10 significantly enriched KEGG pathways and the ordinate indicates the significance of enriched KEGG pathways, the more left, the more significant.

The functionally annotated DEPPs were classified using KEGG pathway enrichment analysis (Figure 5). In both the PI6/UI and PI36/UI comparisons, the top three enriched signaling pathways were focal adhesion, regulation of actin cytoskeleton, and FoxO signaling pathways, while in the PI36/PI6 comparison, adherens junctions, the ErbB signaling pathway, and herpes simplex infection were enriched.

Moreover, some signaling pathways associated with cytoskeleton regulation were enriched in all three comparisons, including regulation of actin cytoskeleton and focal adhesion, indicating that cytoskeletal changes run through the whole process of *E. tenella* sporozoite infection. The mitogen-activated protein kinase (MAPK) and ErbB signaling pathways were also enriched in the PI36/UI comparison, which play important roles in cell signal transduction, transcription, inflammation, and apoptosis.

### PRM results

We selected eight DEPPs for PRM validation from the TMT screening results. Skyline was used to analyze the target peptides, which included the information of chromatographic peaks, original peak area, and the contrast histogram of the original peak area. The results showed that the changes of phosphorylation levels of Zfp36l1, TGFB1I1, MKI67, GIGYF2, and ANLN in all comparisons were consistent with the TMT results, while the changes of phosphorylation levels of VIM, PDCD4, and MAP1B in some comparisons were slightly different from the TMT results. This is because TMT is large-scale screening of non-target proteins; there is a certain false positive rate, so there may be some differences with the PRM results. In conclusion, our TMT results are reliable and reproducible (Table 2).

**Table 2:** PRM analysis and comparison of the quantitative results for candidate phosphoproteins.

Accession	Gene name	Sequence	TMT			PRM		
			6/N	36/N	36/6	6/N	36/N	36/6
S5TQ07	Zfp36l1	SFSEGGER	1.66	3.75	2.25	2.81	7.53	2.68
J08	VIM	DGQVINETSQHHDDLE	1.38	1.20	0.87	1.14	0.96	0.84
1NB68	TGFB1I1	DGLSSPSPR	1.43	1.43	1.00	1.49	1.57	1.06
R4GLV4	MKI67	SEPAEVLSGIK	1.54	0.95	0.62	1.64	0.56	0.34
F1NIY3	PDCD4	FVSEGDGGR	1.55	1.69	1.09	0.98	2.12	2.16
E1C1Q2	MAP1B	SPGDTDYSHDVIGK	1.24	0.95	0.77	0.94	0.75	0.79
A0A1D5PV32	GIGYF2	SQSWEER	1.52	1.98	1.30	1.57	1.64	1.04
A0A1D5P894	ANLN	LSPLQSK	1.28	0.96	0.75	1.08	0.63	0.58

## Discussion

Several studies have shown that a large number of host phosphoproteins are involved in the interaction between parasites and host cells. The analysis and identification of key molecules related to the invasion of host cells by *E. tenella* is of positive significance for elucidating the molecular mechanism of invasion and infection of *E. tenella* and effectively controlling coccidiosis. In this study, we screened DEPPs of DF-1 cells infected with *E. tenella* sporozoites at 6 h and 36 h by TMT-labeled quantitative proteomic technology combined with LC-MS/MS. In total, 10,122 phosphopeptides were identified, matching to 3,398 host phosphoproteins. These DEPPs are mainly involved in cytoskeleton regulation, apoptosis regulation, the immune response, and substance metabolism.

It has been found that many apicomplexans, such as *T. gondii*, *Plasmodium*, and *Cryptosporidium*, remodel the host cytoskeleton in the process of host cell invasion [13, 14, 19, 33, 41, 48]. In the present study, we found that a large number of DEPPs are involved in regulation of the actin cytoskeleton. For example, the phosphorylation level of Rho GTPase activating protein was upregulated at 6 h after infection, and actin-depolymerizing factor, focal adhesion kinase 1, and Gelsolin were only upregulated at 36 h after infection. However, the phosphorylation levels of vimentin, vinculin, paxillin, and LIM domain kinase 1 were upregulated at 6 h and 36 h after infection. Vimentin is an important component of the cytoskeleton; phosphorylation can induce disintegration and spatial orientation alterations of vimentin, thereby regulating cell contraction and local adhesion kinetics [40]. Vinculin can be activated by phosphorylation and plays a role in binding and rearrangement of the actin cytoskeleton [1]. Rho GTPase is a key regulator of cytoskeletal dynamics and affects many cellular processes [20]. Phosphorylation inhibits the binding of GTP to Rac1 and reduces its overall activity [28]. Therefore, we speculate that sporozoites may regulate the cytoskeleton in many ways to facilitate its invasion and intracellular development.

Previous studies have shown that parasites can regulate apoptosis of host cells and create favorable conditions for their parasitism in cells [7, 12, 21, 27]. We found that the phosphorylation level of apoptosis inhibitor 5 (API-5) after 36 h of sporozoite infection was significantly higher than after 6 h and in the uninfected group. Studies have shown that the API-5 phosphorylation level is negatively correlated with the apoptosis rate [37, 38]. Furthermore, the phosphorylation level of apoptosis-antagonizing transcription factor (AATF) after 36 h of sporozoite infection was significantly higher than that of the uninfected group. AATF is a key regulatory factor in the p53 response, and phosphorylation causes AATF to be released from the cytoplasm of MRLC3 and bind to the promoter regions of PUMA, Bax, and BAK, while inhibiting the expression of p53 driven pro apoptotic genes [22]. At 36 h after sporozoite infection, the parasite is at the trophozoite developmental stage and needs the host to provide a suitable development site. Therefore, we speculate that the sporozoite may inhibit apoptosis by regulating the activity of apoptosis-related proteins to facilitate its intracellular development.

Parasites can escape host immune attacks through a variety of immune escape mechanisms. Some studies have found that ATG5 expression was significantly downregulated after *E. tenella* sporozoites infect CEF cells and that ATG5 plays an important role in the formation of autophagy vesicles and has proapoptotic activity [44, 46]. In our study, the phosphorylation level of Atg13 after 36 h of sporozoite infection was 1.685 times that of the uninfected group and 1.309 times that at 6 h after infection. Phosphorylation of Atg13 can prevent its binding to Atg1, while tight Atg1–Atg13 binding is required for autophagic activity [26]. It is suggested that sporozoites may escape immune clearance by interfering with host autophagy to facilitate their invasion and intracellular development.

In addition, host cells also fight parasitic infections through a variety of immune regulatory mechanisms and immune-related proteins [30, 35]. In the present study, the phosphorylation levels of proteins associated with MAPK pathways, such as MAPK1, MAPK11, and MAPK14, were significantly increased. MAPK proteins are mainly transmitters of signals from the cell surface to the nucleus and have important regulatory roles in the production of inflammatory mediators [34]. At 6 h after infection, the expression of Akirin2, a nuclear protein that plays an important role in the innate immune response and embryonic development [15, 32, 42], was 1.6 times higher than in the uninfected group. The transcription factor AP-1 was upregulated at 6 h and 36 h after infection. It is suggested that host cells activate multiple transcription factors such as AP-1 to mediate inflammation initiated by *E. tenella* infection.

All obligate intracellular parasites face the great challenge of getting nutrients from host cells. *E. falciformis* [24] and *T. gondii* [2] can widely affect host cell metabolic pathways or change gene expression patterns. *Plasmodium* are completely dependent on host cell glucose to obtain energy and actively internalize phospholipids from the erythrocyte membrane and the extracellular medium [16, 25]. We found that at 6 h and 36 h after sporozoite infection, the phosphorylation level of 6-phosphofructokinase was 1.4 and 1.5 times higher than in the uninfected group. This enzyme catalyzes the conversion of fructose 6-phosphate to fructose 1,6-diphosphate, which is one of the rate-limiting steps of glycolysis. Phosphorylation changes the tetramer conformation of 6-phosphofructokinase, making its structure stable and preventing its activity from being inhibited by ATP [5]. Furthermore, the increased affinity of phosphorylated 6-phosphofructokinase to F-actin prevents the inhibitory effect of lactic acid on fructose 6-phosphofructokinase and improves glycolysis efficiency [8, 10]. The changes in phosphorylation levels of metabolic-related enzymes suggest that sporozoites may ensure adequate nutritional supply during parasitism by regulating the activity of metabolic pathway-related enzymes.

## Conclusion

In this study, we analyzed the DEPPs between DF-1 cells infected with *E. tenella* sporozoites and uninfected cells, identifying a total of 3,398 differentially expressed phosphoproteins. Our results suggest that the host cytoskeleton may be remodeled during *E. tenella* sporozoite infection, while parasites also evade immune clearance by regulating host apoptosis and interfering with host autophagy. This study provides relevant data and new directions for further exploring the interactions between parasites and host cells.

## Supplementary Material

The supplementary material of this article is available at https://www.parasite-journal.org/10.1051/parasite/2024027/olm. *Table S1*. Details list of proteins of DF-1 cells infected with *Eimeria tenella.* *Table S2*. Details list of phosphorylation peptides of DF-1 cells infected with *Eimeria tenella.* *Table S3*. Details list of significantly regulated phosphopeptides of DF-1 cells infected with *Eimeria tenella.*
